# Upregulated Expression of SOX4 Is Associated with Tumor Growth and Metastasis in Nasopharyngeal Carcinoma

**DOI:** 10.1155/2015/658141

**Published:** 2015-10-22

**Authors:** Si Shi, Xiaolei Cao, Miao Gu, Bo You, Ying Shan, Yiwen You

**Affiliations:** ^1^Department of Otorhinolaryngology Head and Neck Surgery, Affiliated Hospital of Nantong University, Nantong, Jiangsu 226000, China; ^2^Department of Pathology, Medical School of Nantong University, Nantong, Jiangsu, China

## Abstract

SOX4, which belongs to the sex-determining region Y-related high-mobility group (SRY) box family, plays a critical role in embryonic development, cell fate decision, differentiation, and tumor development. Nasopharyngeal carcinoma (NPC) is one of the most common cancers in China and Southeast Asia. However, the molecular mechanisms of this disease remain unknown. In the present study, we used immunohistochemistry to investigate the correlation between the expression of SOX4 with clinicopathologic variables as well as patients prognosis of NPC. We found overexpression of SOX4 was correlated with clinical stages, lymph node metastasis, and Ki-67 expression in NPC (*P* < 0.05). Besides, patients who expressed higher levels of SOX4 had poorer survival rate (*P* < 0.05). Then, in vitro studies, we took serum starvation-refeeding experiment and knocked down the expression of SOX4 with siRNA to demonstrate that SOX4 could promote proliferation of NPC nonkeratinizing cell line CNE2. The regulation of SOX4 on cell migration was determined by the transwell migration assay and wounding healing assay. Besides, we also found SOX4 could promote epithelial-mesenchymal transition (EMT) of CNE2 cells and decrease their cisplatin sensitivity. Our data suggested that SOX4 might play an important role in regulating NPC progression and would provide a potential therapeutic strategy for NPC.

## 1. Introduction

Nasopharyngeal carcinoma (NPC) is a solid tumor arising from the epithelial cells of the nasopharynx. Although it is a rare disease in North America and Europe, it is common in endemic areas including the southern part of China, Southeast Asia, and North Africa [1]. Major etiologic factors of NPC include genetic susceptibility, environmental factors, and Epstein-Barr virus (EBV) infection [2]. Radiotherapy and adjuvant chemotherapy are the main treatments for NPC patients and the survival rate has improved with advances in therapy [3]. However, NPC has characteristics of early cervical lymph node metastasis and distant metastasis. Once metastasis occurs, the prognosis may be poor [4, 5]. Thus, better understanding of the molecular mechanisms underlying NPC progression is essential for the early diagnosis and the development of effective therapeutic agents.

SOX4, a 47-kDa protein member of SOX family, is encoded by a single exon gene [6]. It belongs to the highly conserved group C SOX and may be distinguished from other SOX transcription factors by the conserved C-terminal transactivation domain (TAD) [7, 8]. Like other SOX family members, SOX4 can interact with other transcription factors and play a significant part in embryonic development, including the development of the central and peripheral nervous system, thymocytes differentiation, and embryonic cardiac and osteoblastic differentiation [7–9]. In addition, as SOX4 plays a role in controlling cell fate and differentiation, aberrant regulation of SOX4 can be associated with tumorigenesis. Several evidences have showed SOX4 expression may inhibit cell apoptosis, increase cell invasion and metastasis, and maintain cancer-initiating cells [8]. SOX4 has been demonstrated to be highly expressed in breast cancer, lung cancer, glioma, prostate cancer, and gastric cancer [10–14]. However, in contrast to the tumor-promoting role of SOX4, it can also act as a suppressor in some tumors, including bladder carcinoma, melanoma, and gallbladder cancer [15–17]. SOX4 promotes cell cycle arrest and apoptosis to inhibit tumorigenesis in these tumors. The outcome of SOX4 activation depends on the cellular context and the tumor origin [8].

Nevertheless, whether SOX4 may impact the progression of NPC has not been detected. In the present study, we investigated the involvement of SOX4 in NPC, which might help us have a better understanding of the molecular mechanism. First, we demonstrated that SOX4 was remarkably upregulated in NPC tissues and cell line. Then, we evaluated its associations with clinical and pathologic factors. To further study the roles of SOX4 in NPC, we knocked down its expression using anti-SOX4 small-interfering RNA (siRNA) and found SOX4 was associated with growth and metastasis of NPC cell line CNE2. At last, we confirmed underexpression of SOX4 could induce chemosensitivity of CNE2 cells. All the data suggested that SOX4 might be a positive regulator in NPC progression.

## 2. Materials and Methods

### 2.1. Patients and Tissue Samples

A total of 55 NPC specimens were obtained from the Affiliated Hospital of Nantong University from 2009 to 2013. All patients had not received any therapies such as radiation or chemotherapy at the time of original biopsy. Chronic inflammatory nasopharyngeal epithelium tissues were used as control. The main clinical and pathologic variables were shown in Table 1 and the follow-up data were available for all patients. The clinical processes were approval from the Ethics Committee of Affiliated Hospital of Nantong University and every patient had written informed consent. Fresh samples were frozen in liquid nitrogen and then maintained at −80°C until use for protein extraction. Some tissue samples were immediately fixed in formalin and embedded in wax for immunohistochemistry. The pathological diagnoses of the specimens were according to the 2005 WHO histological classification and the pathologic stage was according to the UICC 1997 staging system of NPC.

### 2.2. Immunohistochemistry

NPC biopsy specimens and normal biopsy specimens were fixed with formalin, embedded in paraffin, and then sectioned at 4 mm thickness. The sections were rehydrated in graded ethanol. Endogenous peroxidase activity was inactivated by soaking in 0.3% hydrogen peroxide for 30 min. Then, the sections were processed in 10 mmol/L citrate buffer (pH = 6.0) and heated to 121°C in an autoclave for 20 min to retrieve the antigen. After being rinsed in phosphate buffered saline (PBS), 10% goat serum was applied for 1 h at room temperature to block any nonspecific reactions. The sections were then incubated with anti-SOX4 antibody (diluted 1 : 100, Abcam) and anti-Ki-67 antibody (diluted 1 : 100, Santa Cruz Biotechnology) overnight at 4°C. Negative control slides were processed as the primary antibody using a nonspecific immunoglobulin IgG (diluted 1 : 100, Santa Cruz Biotechnology) at the same concentration. Then, the slides were rinsed with PBS and processed using the peroxidase-anti-peroxidase method (DAKO, Hamburg, Germany) according to the manufacturer's instructions. Color reaction was developed by using 3,39-diaminobenzidine tetrachloride (DAB) chromogen solution. All the sections were counterstained with hematoxylin, dehydrated, and cover-slipped.

The immunohistochemistry scoring results were evaluated independently by two pathologists without knowing the patient's clinicopathological outcomes and then analyzed according to the intensity of the staining and the relative abundance of positive cells. Five high-power (200x magnification) fields were chosen randomly for each section, and 500 cells were counted per field. The intensity of SOX4 nuclear staining was scored as 0 (no staining), 1 (weak), 2 (medium), and 3 (strong). The abundance of positive cells was graded as 0 (<10% positive cells), 1 (10–25%), 2 (26–75%), and 3 (76–100%). The expression level of SOX4 was defined by the sum of the staining-intensity and staining-extent scores as follows: “−” (negative, score of 0), “+” (weakly positive, score of 1-2), “++” (positive, score of 3-4), and “+++” (strongly positive, score of 5-6) [18]. “−” and “+” were defined as low expression, and “++” and “+++” were defined as high expression. As for statistical analysis of Ki-67 stain, a cut-off value of 50% or more positively stained nuclei in five high-power fields was used to define Ki-67 staining: low expression group (<50%) and high expression group (≥50%).

### 2.3. Cell Culture and Cell Cycle Analysis

Nasopharyngeal carcinoma nonkeratinizing cell line CNE2 and the immortalized normal nasopharyngeal epithelial cell line NP69 were generously given by Sun Yat-sen University Cancer Center. CNE2 cells were cultured in RPMI medium 1640 (Gibco BRL, Grand Island, NY) supplemented with 10% fetal bovine serum (FBS) (Gibco), while NP69 cells were grown in Keratinocyte-SFM medium supplemented with epidermal growth factor (EGF) (Invitrogen, Carlsbad, USA). All cell lines were incubated at 37°C in 5% CO_2_. For cell cycle analysis, harvested cells were rinsed with cold PBS and fixed in 70% ethanol at 4°C overnight and then incubated with 1 mg/mL RNase A for 30 min at 37°C. After that, cells were stained with propidium iodide (50 *μ*g/mL PI) (Becton Dickinson, San Jose, CA) in PBS with 0.5% Tween-20. The DNA content of labeled cells was analyzed using a Becton Dickinson flow cytometer BD FACSCAN (Becton Dickinson, San Jose, CA), Cell Quest acquisition and analysis programs.

### 2.4. RNA Isolation and Quantitative RT-PCR

Total RNA was extracted using the Trizol reagent (Sigma, USA) from CNE2 and NP69 according to the manufacturer's instructions and was reverse transcribed into cDNA samples using a Transcriptor First Strand cDNA Synthesis Kit (Roche, Germany, 04 896 866 001). Gene expression was analyzed using Taqman Universal PCR Master Mix. GAPDH was used to normalize the relative expression levels of genes. The primers used were obtained from Biomics Biotechnologies Co., Ltd. (Nantong, China) and were as follows: SOX4 forward: CACTCCTCCTCTTCCTCCTC; reverse: GCCGACGACGAACTGAAG and GAPDH forward: forward: 5′-GAAGGTGAAGGTCGGAGTC-3′; reverse: 5′-GAAGATGGTGATGGGATTTC-3′. The amplification conditions of PCR were 30 min at 42°C for reverse transcription, one cycle at 94°C for 2 min, 35 cycles at 94°C for 20 s, 58°C for 20 s, and 72°C for 30 s. The results were normalized with GAPDH. The Ct-value was calculated with the ΔΔCt method and expressed as 2^−ΔΔCt^. All the reactions were performed in triplicate.

### 2.5. Western Blotting

Cells and tissues were washed twice with ice-cold PBS and lysed using RIPA Lysis Buffer. The concentration of total protein was measured by BCA Protein Assay Kit and 20 *μ*g of total cellular protein was separated by 10% sodium dodecyl sulfate-polyacrylamide gel electrophoresis (SDS-PAGE). After electrophoresis, proteins were transferred to polyvinylidene difluoride filter (PVDF) membranes (Millipore, Bedford, MA) and then blocked with 5% nonfat milk in TBST for 2 h. The membrane was incubated with the indicated primary anti-SOX4 (1 : 1000, Abcam) overnight at 4°C; then, it was incubated with HRP-tagged secondary antibodies (1 : 1000, Santa Cruz Biotechnology) at room temperature for 1.5 hours. After washing with TBST for three times, ECL reagent (Millipore) was used to visualize the immune complexes. ImageJ was used to compare the band density and we took *β*-actin (1 : 2000, Santa Cruz Biotechnology) as a loading control. All the experiments were carried out on three separate occasions.

### 2.6. Transient Transfection with siRNAs

Small-interfering RNA (siRNA) and silencer negative control-siRNA (snc-RNA) of SOX4 were obtained from Biomics Biotechnologies Co. Ltd. (Nantong, China). CNE2 cells were plated onto a 6-well plate or a 96-well plate (Beyotime, Biotech, China) and grown to 30–50% confluence. Then, they were transfected with SOX4-siRNA or control siRNA by Lipofectamine 2000 (Invitrogen, USA) according to the manufacturer's instructions. Transfected cells were used for the experiments 48 h or 72 h after transfection.

### 2.7. Cell Proliferation Assay

The cell viability rate of CNE2 cells was assessed using cell counting kit-8 (CCK-8 Kit, Beyotime Institute of Biotechnology). CNE2 cells transfected with SOX4-siRNA or control-siRNA were seeded onto 96-well plate (Corning Inc., Corning, NY) at a density of 1 × 10^4^ cells per well with 100 *μ*L RPMI-1640 and grown overnight in a 5% CO_2_, 37°C atmosphere. Then, the cells were washed with PBS, at time points of 0 h, 6 h, 12 h, 24 h, 36 h, and 48 h, and 10 *μ*L CCK-8 solution was added to each well and incubated for another 1.5 hours. The absorbance of each well was measured at 450 nm using a microplate reader. Five wells were used for each experimental condition and all the experiments were independently repeated 3 times.

### 2.8. Wound-Healing Assay

CNE2 cells transfected with either SOX4-siRNA or control-siRNA were seeded on 6-well plates at a density of 5 × 10^5^ cells/well. After cells confluence reached about 80%, the monolayer cells were wounded by scraping off the cells using a 100 *μ*L pipette tip. Then, the cells were washed with PBS to remove free-floating cells and debris. The culture plates were incubated at 37°C. Wound healing was observed at different time points, and the migration distance of cells was imaged under a microscope. The relative distance of cells was measured by the wound width/the distance measured at 0 h.

### 2.9. Transwell Migration Assay

Cell migration was determined using a Millipore chamber containing a polycarbonate filter with an 8 mm pore size (Millipore). 1 × 10^5^ CNE2 cells transfected with either SOX4-siRNA or control-siRNA were resuspended in 200 *μ*L serum-free 1640 and added to the upper chamber, while 500 *μ*L RPMI 1640 with 10% FBS was added to the lower chamber. After 16 h incubation at 37°C, the cells in the upper chamber were carefully removed with a cotton swab and the reverse side of the upper chamber was washed with PBS. Cells adhering to the lower surface were fixed in 100% methanol for 30 minutes and stained with crystal violet; the cells were then visualized under a microscope. Cell number was quantitated using SPSS software.

### 2.10. Statistical Analysis

All the data were independently repeated 3 times and reported as the means ± standard deviation (SD). Statistical analysis was performed using SPSS17.0 software. The associations among the SOX4 expression, Ki-67 expression, and clinicopathological features were analyzed using the *χ* ^2^ test. For analysis of survival data, Kaplan-Meier curves were constructed, and the log-rank test was performed. Multivariate analysis was performed using Cox's proportional hazards model. The *P* value was based on the two-sided statistical analysis and *P* value less than 0.05 was considered statistically significant.

## 3. Results

### 3.1. The Expression of SOX4 in NPC

**Figure 1:**
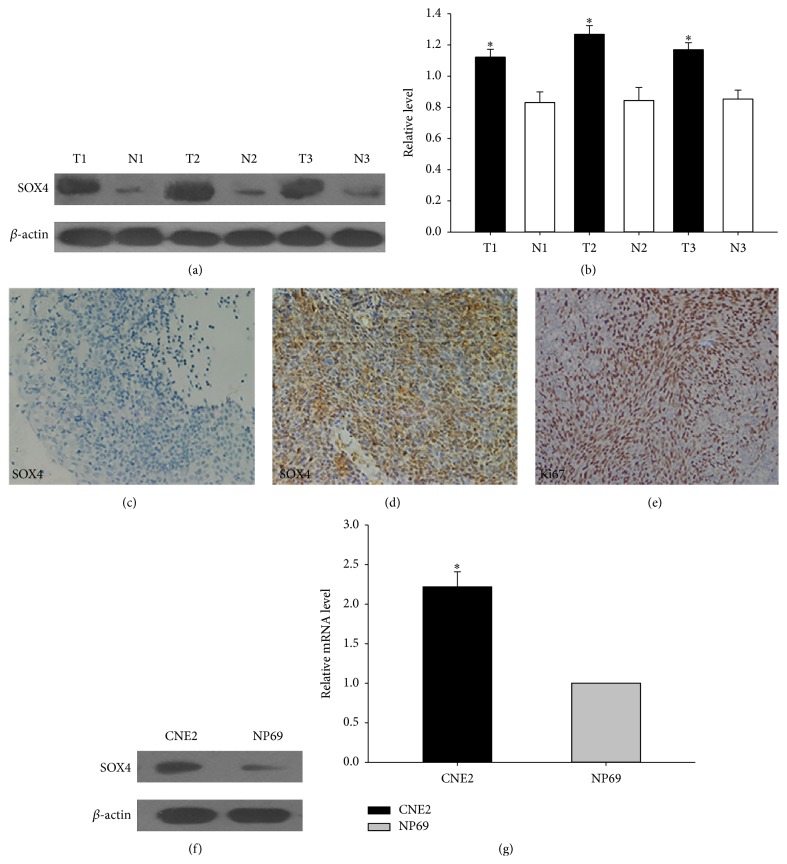
The expression of SOX4 in NPC. (a) Western blot was performed to detect the expression of SOX4 in 3 NPC tissues and 3 inflammatory nasopharyngeal tissues. (T) Nasopharyngeal squamous cell carcinoma tissues. (N) Inflammatory nasopharyngeal epithelium tissues. *β*-actin was used as a control. (b) The bar demonstrated the ratio of SOX4 protein expression to *β*-actin by densitometry. (c) Negative staining of SOX4 in the control inflammatory nasopharyngeal epithelial tissues (×200). (d) High expressions of SOX4 were observed in NPC tissues (×200). (e) High expressions of Ki67 were observed in NPC tissues (×200). (f) Western blot analysis of SOX4 expression in CNE2 and NP69. (g) qRT-PCR was used to detect the relative expression of SOX4 in two cell lines. The data shown were representative of at least three independent experiments. ^*∗*^ *P* < 0.05.

To determine whether the level of SOX4 was associated with NPC, we first examined the expression of SOX4 in 3 pairs of NPC tissues and the inflammatory nasopharyngeal epithelium tissues by western blot analysis. As shown in Figures 1(a) and 1(b), SOX4 was highly expressed in NPC tissues. Then, we performed immunohistochemistry assay to further confirm the specificity of the results above. The expression of SOX4 was detected in 55 NPC specimens. In most NPC specimens, immunoreactivity of SOX4 was seen both in the cytoplasm and nuclei, but predominantly in the nuclei. Most importantly, the expression level of SOX4 was high in NPC tissues (Figure 1(d)). However, Figure 1(c) showed SOX4 was hardly expressed in inflammatory nasopharyngeal epithelium tissues. We also detected the expression of the proliferation index Ki67. It was scored as high expression with strong nuclei staining (Figure 1(e)). SOX4 expression was also shown in cell lines at protein and mRNA levels (Figures 1(f) and 1(g)). Because of its higher expression in NPC tissues and cell line, we hypothesized it might play an important role in NPC.

### 3.2. Correlation of SOX4 Expression with Clinicopathologic Variables in NPC

To further determine the clinical significance of SOX4 in NPC, we explored the relationship between clinicopathologic variables with SOX4 expression. The clinicopathological data of the patients were summarized in Table 1 and the carcinoma specimens were divided into high expressors and low expressors. As shown in Table 1, there were significant positive correlations between the expression of SOX4 and Ki-67 (*P* < 0.05). Besides, SOX4 expression level was also associated with clinical stages and lymph node metastasis (*P* < 0.05). However, it did not correlate significantly with gender and age (*P* > 0.05).

### 3.3. High Expression of SOX4 Predicted Poor Prognosis of NPC Patients

**Figure 2:**
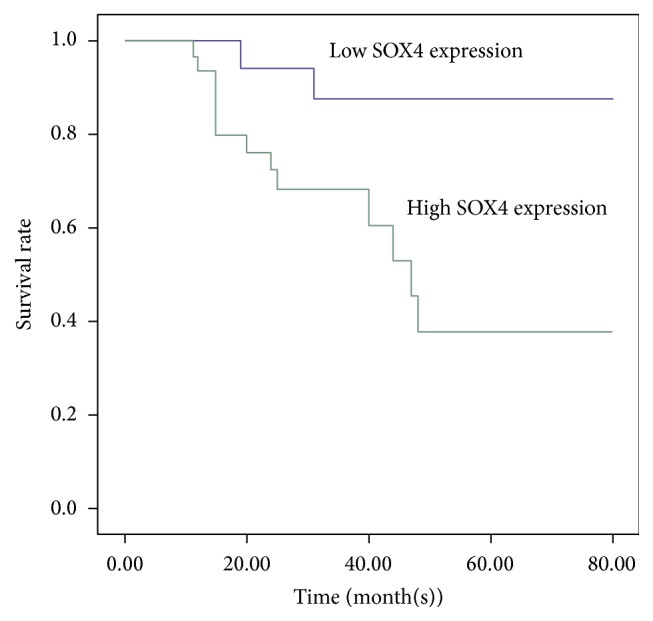
The expression of SOX4 predicted the prognosis of NPC patients. Kaplan-Meier survival curves of NPC patients were based on SOX4 expression status. Patients had higher SOX4 expression versus lower SOX4 expression which showed highly significant separation. ^*∗*^ *P* < 0.05.

Survival analysis was restricted to 55 patients with follow-up data and immunohistochemistry results. Using Kaplan-Meier analysis, we found high expression of SOX4 was associated with decreased overall survival of NPC (Figure 2; *P* < 0.05). Moreover, we used multivariate analysis to determine whether SOX4 was the potential prognostic factor for NPC. The results showed SOX4 expression and clinical stage (Table 2; *P* < 0.05) were independent prognostic indicators for patients' overall survival.

### 3.4. SOX4 Was Highly Expressed in Proliferating CNE2 Cells

**Figure 3:**
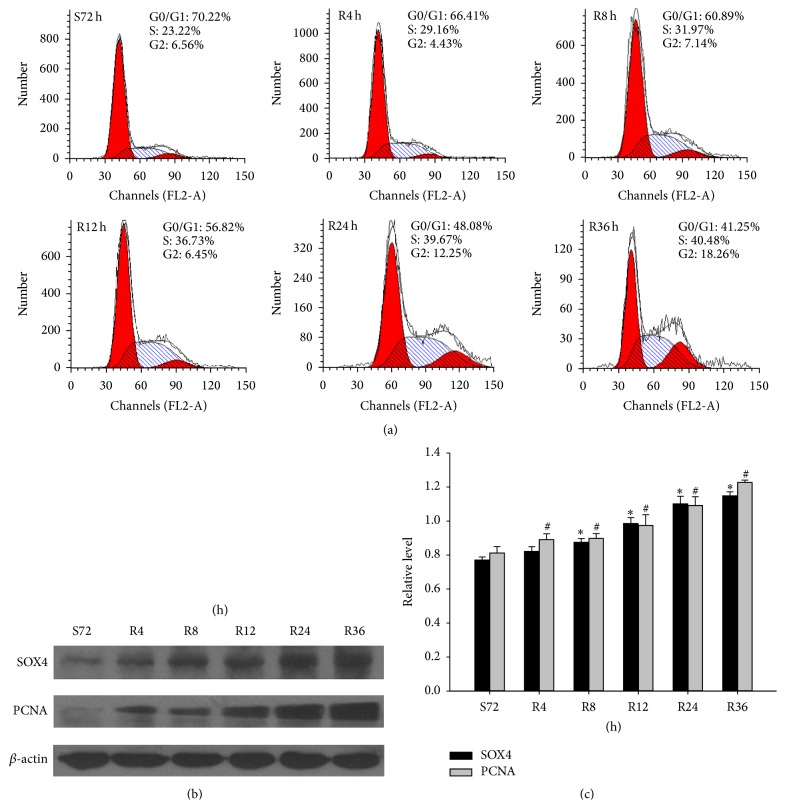
The expression of SOX4 in proliferating CNE2 cells. (a) Flow cytometry was used to quantify the cell cycle progress in CNE2 cells. The cells were subjected to serum starvation for 72 h (S72 h) and then progressed into cell cycle by refeeding medium containing 10% FBS for 4, 8, 12, 24, and 36 h (R4 h, R8 h, R12 h, R24 h, and R36 h). (b) Western blot was used to investigate the expression of SOX4 and PCNA in CNE2 cells that were subjected to serum starvation and refeeding. *β*-actin was used as a control for protein load and integrity. (c) The bar demonstrated the ratio of SOX4 and PCNA protein expression to *β*-actin by densitometry. The data shown were representative of at least three independent experiments. Data were analyzed by Student's *t*-test. ^*∗*#^ *P* < 0.05.

Then, we made serum starvation and refeeding assay to detect the changes of SOX4 during cell cycle progression. CNE2 cells were arrested in G1 phase by serum deprivation for 72 h and the percentage in the G1 phase increased. Then, serum was added; the cells were released from the G1 phase (from 70.22% at S72 h to 41.25% at R36 h) and increased in S phase (from 23.22% at S72 h to 40.48% at R36 h) (Figure 3(a)). Western blot showed that SOX4 increased in CNE2 cells after serum stimulation as early as 4 h. Its expression increased with cell-cycle progression. Meanwhile, PCNA, a general marker of dividing cells, was also upregulated (Figure 3(b)). The results confirmed SOX4 plays an important role in regulating cell proliferation in a cell cycle-dependent pathway.

### 3.5. Interference of SOX4 Expression Inhibited the Proliferation of CNE2 Cells

**Figure 4:**
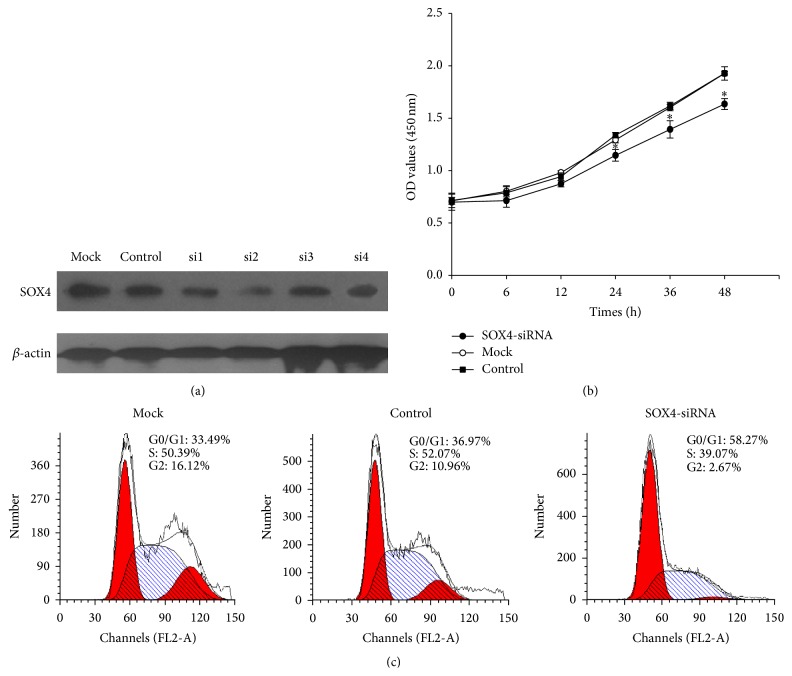
Interference of SOX4 expression suppressed the proliferation of CNE2 cells. (a) CNE2 cells were transiently transfected with siRNA targeting SOX4 (si-1, si-2, si-4, and si-4) or a scrambled sequence (control siRNA). A representative western blot image showed the expression of SOX4. (b) CCK8 assay was used to determine cell viability of CNE2 cells treated with SOX4-siRNA or control siRNA for the indicated time. The data were means ± SEM (^*∗*^ *P* < 0.05). (c) Flow cytometric analysis was used to evaluate cell cycle distribution of CNE2 after being transfected with SOX4-siRNA or control siRNA for 48 h.

To determine the effect of SOX4 on the growth of CNE2 cells, we used transient transfection to downregulate its expression. The knockdown efficiency was confirmed by western blot; the SOX4-si2 reduced the level of protein expression most compared with the control and the mock CNE2 cells (Figure 4(a)). Therefore, we chose SOX4-si2 for the further investigation. CCK8 assay showed that downregulating the expression of SOX4 caused a significant decrease of the proliferation rate in CNE2 cells (Figure 4(b)). To further explore its mechanism, we used flow cytometry analysis to determine the cell cycle distribution. The results showed that the percentage of cells in the G0/G1 phase increased from 36.97% to 58.27% after transfecting with SOX4-siRNA. At the same time, the S phase was obviously decreased from 52.07% to 39.07% (Figure 4(c)). All these data suggested that SOX4 was able to regulate proliferation of CNE2 cells by promoting G0/G1-S transition.

### 3.6. Interference of SOX4 Expression Inhibited the Migration of CNE2 Cells

**Figure 5:**
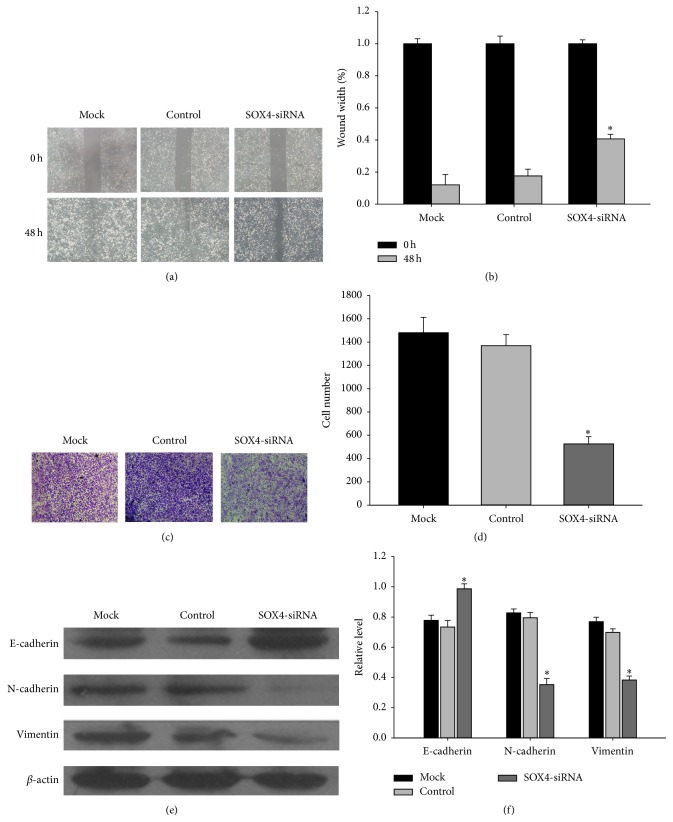
Interference of SOX4 expression inhibited the migration of CNE2 cells. (a) CNE2 cells transfected with SOX4-siRNA or control siRNA were used for the wound-healing assay. Migration of cells to the wound was visualized at 0 and 48 h with a microscope (×200 magnification). (b) The histogram showed the relative wound width. The data are mean ± SEM (^*∗*^ *P* < 0.05). (c) 1 × 10^5^ serum-free CNE2 cells which were transfected with SOX4-siRNA or control siRNA. After 16 h, representative images of the migrated cells were taken at the same magnification. (d) Absolute number of cells migrated through the transwell membrane. The member was counted in 10 fields under ×20 objective lens. (e) Western blot was used to investigate the changes of EMT markers in CNE2 cells transfected with SOX4-siRNA. (f) The bar demonstrated the expression ratio of the target protein to *β*-actin by densitometry. The data shown were representative of at least three independent experiments. Data were analyzed by Student's *t*-test. ^*∗*^ *P* < 0.05.

Tumor metastasis often leads to poor prognosis of NPC, we then investigated the role of SOX4 in NPC metastasis. The effect of SOX4 silencing on migration was investigated by wound-healing assay and transwell migration assay. Following incubation of physically wounded cells for 48 h, the relative wound width was longer in SOX4-siRNA CNE2 cells compared with the control (Figures 5(a) and 5(b)). Similarly, the transwell migration assays showed that more CNE2 cells migrated through the membrane compared with the cells that had lower expression of SOX4 after 16 h (Figures 5(c) and 5(d)).

Some studies have reported that SOX4 is involved in the promotion of epithelial-mesenchymal transition (EMT) during tumor metastasis. Therefore, we wanted to know whether the effects of SOX4 on CNE2 cells migration were associated with EMT. To address the hypothesis, we used western blot analysis. As could be seen, the downregulation of SOX4 was accompanied by the upregulation of epithelial marker E-cadherin and downregulation of mesenchymal marker vimentin and N-cadherin (Figures 5(e) and 5(f)). So SOX4 knockdown inhibited the migration of CNE2 cells and could partially reverse its EMT phenotype.

### 3.7. Downregulation of SOX4 Increased Cisplatin Sensitivity in CNE2 Cells

**Figure 6:**
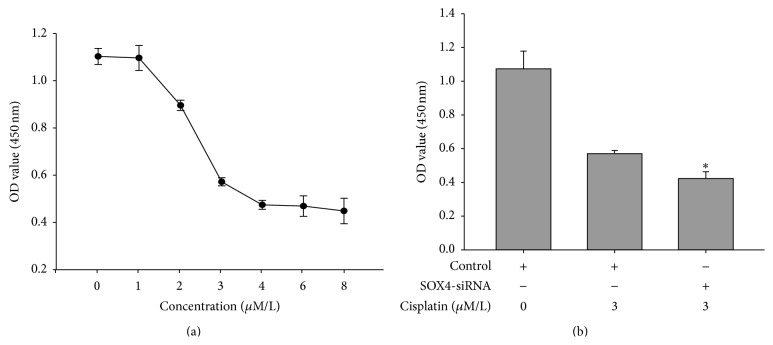
Downregulation of SOX4 increased cisplatin sensitivity in CNE2 cells. (a) CCK8 assay was used to evaluate cell viability after the treatment of cisplatin for 48 h at concentration of 0.1.2.3.4.6.8 *μ*mol/L, respectively. The data are mean ± SEM (^*∗*^ *P* < 0.05). (b) CNE2 cells were transfected with either SOX4-siRNA or control siRNA and then subjected to treatment with or without cisplatin for 48 h. CCK-8 assay was used to measure cell viability. The data shown were representative of at least three independent experiments. Data were analyzed by Student's *t*-test. ^*∗*^ *P* < 0.05.

Cisplatin is one of the chemotherapeutic agents in the treatment of NPC. We wanted to analyze if cisplatin-mediated growth inhibition of NPC cell line CNE2 could be modified by the expression of SOX4. First, we determined the concentration of cisplatin to inhibit 50% CNE2 growth. As shown in Figure 6(a), CNE2 was sensitive to cisplatin with decreasing of cell viability in a dose-dependent manner and the IC50 (inhibitory concentration 50) was about 3 *μ*mol/L. Then, we tested whether SOX4 affected the response of CNE2 cells to cisplatin treatment. The cells were transfected with SOX4-siRNA and treated with cisplatin in a concentration of 3 *μ*mol/L for 48 h. CCK8 assay indicated that the survival rate of the transfected cells was obviously lower than control (Figure 6(b)). Thus, we confirmed knocking down the expression of SOX4 might increase the cisplatin sensitivity of CNE2 cells.

## 4. Discussion

NPC is one of the most common cancers in China and Southeast Asia [19]. The prognosis may be poor because of local recurrence and distant metastasis [20]. However, the molecular mechanisms of NPC are still poorly understood. Nowadays, more and more studies have reported that genetic alterations play a vital role in tumor progression, so the studies of novel molecules that have changed during NPC progression may help us find prognostic markers and therapeutic targets. In the present study, we tried to investigate the role of SOX4 in the progression of NPC.

Some studies have revealed that SOX4 exerted specific oncogenic function in tumors, including lung, breast, bladder, prostate, and endometrium, while others reported that SOX4 was downregulated in some tumors and suppressed the progression. This discrepancy could be due to the gene mutations, miRNAs, various signaling pathways, and intrinsic differences in the biology of various types of tissues [16]. Whether SOX4 acted as a suppressor gene or not in NPC was the main emphasis in our research.

In this study, we first assessed the expression levels of SOX4 in 3 pairs of clinical fresh tissues, 55 cases of clinical paraffin-embedded NPC tissues, and NPC cell line CNE2. We found that the expression of SOX4 was significantly higher in NPC samples compared with inflammatory nasopharyngeal epithelium tissues. Immunostaining of SOX4 was mainly localized in the nuclei, and it was positively associated with Ki-67 (Figure 1). Precise prediction of prognosis may be important for us to take further therapy for tumor patients. In prostate cancer, gastric cancer, and colon cancer, SOX4 might be used as a marker to predict the prognosis [13, 14, 21]. In our research, SOX4 expression level was also associated with clinical stage and lymph node metastasis of NPC and correlated with patients' overall survival time (Tables 1 and 2; Figure 2). The results above were consistent with previous studies. All these indicated that SOX4 might be associated with the progression of NPC and act as a clinical biomarker for evaluating the prognosis.

These results promoted us to further explore the function of SOX4 in vitro. We detected its expression during cell cycle progression in CNE2 cells and found that SOX4 increased during the G1 to S phase, this confirmed the connection between SOX4 overexpression and cancer progression (Figure 3). Then, we knocked down SOX4 in CNE2 cells by siRNA and it was accompanied with a significant decrease of cell proliferation rate. This indicated SOX4 could regulate the proliferation of CNE2 cells and might partly explain the association of SOX4 with Ki67 in NPC samples (Figure 4(b)). Next we investigated the mechanism underlying the decreased cell growth rate. Flow cytometry analysis showed SOX4 could promote proliferation of CNE2 cells through regulating cell cycle progression (Figure 4(c)).

As metastasis is a biological character which influences NPC patients' prognosis [22], we defined a new role for SOX4 as a regulator of NPC metastasis. Our conclusion was based on the observation that silencing the expression of SOX4 might inhibit the migrating ability of CNE2 (Figures 5(a) and 5(c)).

Although SOX4 has been reported to play an important role in regulating cell migration in several tumor types, the mechanisms need to be lucubrated. In some cancers, the expression of SOX4 mRNA was particularly elevated in metastases and this was associated with the recurrence and prognosis of these tumor types [23]. Epithelial-mesenchymal transition (EMT) is a process where cells convert from the epithelial to the mesenchymal state. When EMT occurs, cells have characteristics such as loss of cell-cell contacts, apical-basal polarity, and acquisition of migratory and invasive properties [24]. Previous studies had proved that SOX4 was involved in the promotion of EMT. Zhang et al. showed that overexpression of SOX4 in human mammary epithelial cells contributed to invasion and metastasis of breast cancer cells, which was associated with activating the TGF-*β* pathway to induce EMT [10]. In HCC, silencing the expression of SOX4 might decrease the mesenchymal markers of HCC cells [23]. Wang et al. also confirmed that SOX4 could promote EMT in prostate cancer [25]. All these demonstrated the role of SOX4 in tumor during EMT. In the present study, we found that SOX4 might act as a trigger for EMT in NPC. Knocking down SOX4 in CNE2 cells could repress mesenchymal markers expression (Figure 5(e)). Taken together, these results provided evidence that SOX4 could function as a supporter in metastasis of NPC.

Cisplatin is a potent chemotherapeutic agent in the treatment of human malignancies, including NPC. Studies had proved that combination of cisplatin-based chemotherapy with radiation could improve 5-year overall survival rate and 5-year disease-free survival rate in NPC [26]. However, because of the variation in treatment responses and chemoresistance, there were still many patients who do not benefit from the current therapy [27]. This made us explore the role of SOX4 in cisplatin-mediated growth inhibition. The results showed that SOX4-siRNA could promote cell death in the treatment of cisplatin. So the expression of SOX4 might decrease the cisplatin sensitivity of CNE2 cells (Figure 6).

## 5. Conclusion

Our study indicated that SOX4 expression could provide important prognostic information of NPC. Besides, it was associated with CNE2 cells growth, metastasis, and the sensitivity to cisplatin. Therefore, SOX4 may serve as a novel molecular target for the detection and treatment of NPC.

## Figures and Tables

**Table 1:** The association between the expression of SOX4 and clinicopathological parameters of NPC.

Clinicopathological parameters	Total	SOX4 expression		*P*
		Low	High	
Gender	55			
Male	43	17	26	0.574
Female	12	5	7	
Age (year)				
<50	14	5	9	0.762
⩾50	41	17	24	
Clinical stages				
I	2	2	0	0.030^*∗*^
II	8	6	2	
III	29	10	19	
IV	16	4	12	
Lymph node metastasis				
yes	37	11	26	0.040^*∗*^
no	18	11	7	
Ki-67 expression				
low	25	16	9	0.002^*∗*^
high	30	6	24	

Statistical analyses were performed by the Pearson *χ* ^2^ test. ^*∗*^ *P* < 0.05 was considered significant.

**Table 2:** Contribution of various potential prognostic factors to survival by Cox regression analysis on 55 NPC samples.

	Hazard ratio	*P*	95.0% confidence interval
Age	1.381	0.602	0.410–4.650
Gender	0.425	0.185	0.120–1.506
Lymph node metastasis	1.484	0.156	0.662–13.030
Clinical stage	3.012	0.011^*∗*^	1.292–7.020
SOX4 expression	3.608	0.027^*∗*^	1.208–24.027

Statistical analyses were performed by the Cox regression analysis. ^*∗*^ *P* < 0.05 was considered significant.
